# Obstacles to birth registration in Niger: estimates from a recent household survey

**DOI:** 10.1186/s41043-019-0185-1

**Published:** 2019-10-18

**Authors:** Quentin Wodon, Ali Yedan

**Affiliations:** World Bank Group, 1818 H Street, NW, Washington, DC, 20433 USA

**Keywords:** Birth registration, Niger, Civil registration and vital statistics (CRVS)

## Abstract

Despite progress made towards increasing birth registration rates over the last dozen years, almost one in two children may still not get registered at birth in Niger according to a recent nationally representative household survey. What can be done to improve birth registration rates? This paper relies on a simple approach to measure how solving various obstacles to birth registration faced by parents could help increase birth registration rates. Controlling for other factors affecting birth registrations, the analysis relies on local-level reasons declared by households for not registering their children. The estimation method provides measures of potential gains in birth registration rates from different actions, including providing services closer to where households live, improving household knowledge about the fact that birth registration is both mandatory and beneficial for children, and reducing the out-of-pocket costs of birth registration. The analysis remains exploratory, but it provides hopefully useful insights about the likely benefits that could be derived from various policies utilized for increasing rates of birth registrations.

## Background

Birth registration is defined as “the continuous, permanent and universal recording within the civil registry of the occurrence and characteristics of birth” [1]. There is broad consensus that birth registration is essential for development given its effect on the ability of children to benefit from educational and health services and outcomes [2] in many countries and more generally for development [3]. Birth registration is widely considered to be an essential intervention for early childhood development [4] as well as a human right, which itself matters for the exercise of other human rights, including civil and political rights, and for preventing violations of other rights, for example, in the case of child marriage [5]. Ensuring universal birth registration is a target under the Sustainable Development Goals (SDG) (target 16.9). Progress towards that target is measured through the proportion of children under 5 years of age whose births have been registered with a civil authority. Unfortunately, in many countries, there is a long way to go [6] because birth registration rates among children under 5 remain low [7], especially in Sub-Saharan Africa [8].

From a management and policy point of view, birth registration is a component of civil registration. Systems for birth registration are therefore part of broader civil registration and vital statistics (CRVS) systems. Civil registration is defined by the United Nations (UN) as the “universal, continuous, permanent and compulsory recording of vital events provided through decree or regulation in accordance with the legal requirements of each country” and vital statistics are “a collection of statistics on vital events in a lifetime of a person as well as relevant characteristics of the events themselves and of the person and persons concerned.” [9] While birth registration systems should ideally be fully integrated into CRVS systems, they often operate as a distinct system across ministries, as is also to some extent the case in Niger.

International agencies such as the United Nations International Children’s Emergency Fund (UNICEF), the World Health Organization (WHO), and the World Bank Group (WBG) are supporting efforts by low- and middle-income countries to improve their CRVS systems [10]. In some cases, these efforts have yielded substantial progress, including in Sub-Saharan African countries where birth registration rates have been traditionally low. Niger is a great example of progress. The country has achieved major gains in increasing birth registration rates [11]. Between the 2006 and 2012 Demographic and Health Surveys, birth registration rates for children under the age of 5 doubled from 32 to 64%. However, as noted by UNICEF [12], the rate of birth registration declined from 65% in 2016 to 53% in 2017 as agents at registration centers were not paid. This led to some of them stopping registering births. Fortunately, the 2018 Law of Finance allocated more funding for civil registration and the government adopted in 2017 a strategic plan to make the civil registration system universal and free. Funding gaps remain to achieve this objective.

Estimates from a 2015 survey used in this paper put birth registration rates at 52% nationally, but regardless of which percentage estimate is used, which may depend on the survey, there is no doubt that major gains have been achieved. By comparison, in the 12 years preceding the 2006 Demographic and Health Surveys, the gains achieved were much smaller, at only 8 percentage points, from 24 to 32%.

How did Niger achieve such gains? As described in the recent diagnostic conducted by the World Bank, the birth registration process became more efficient thanks to the introduction of auxiliary notification offices. Since 2008, the civil registration system has consisted of not only primary and secondary registration centers located in the administrative capitals of communes or communes’ substructures, but also of auxiliary notification offices in healthcare facilities where vital events occur. Staff in these auxiliary centers notify primary or secondary centers of vital events such as births, with these centers then completing the birth registration process and issuing official documents. This helped triple the number of registration centers between 2007 and 2012. In addition, the fact that births at healthcare facilities are now automatically registered reduces leakage. Finally, large mobile birth registration campaigns were implemented in the country between 2009 and 2011. Of these various initiatives, the creation of the auxiliary notification offices is probably the initiative that had the largest impact because it enabled parents and midwives to record births where the children were born.

Despite major progress, much remains to be done. According to the national survey used in this paper, in 2015, almost one in two children was still at risk of not being registered. Guidance is available in the literature on interventions that can help boost birth registration rates [13] as well as best practices [14]. Yet at the country level, there is a benefit in conducting country-specific analysis to better understand constraints leading parents to not register their children. The objective of this paper is to look at the correlations of birth registrations and obstacles that lead some parents to not register their children using regression analysis. Looking at the drivers of birth registration has been done before using various types of methods in several African countries, including Ghana [15], Nigeria [16], Kenya [17], and Zimbabwe [18], but not in the way suggested in this paper.

The paper relies on a simple approach to assess how solving various obstacles to birth registration could help. Controlling for other factors affecting birth registrations, the estimation provides measures of potential gains in birth registration rates from providing services closer to households, improving knowledge among parents about the fact that birth registration is both mandatory and beneficial for children, and reducing the out-of-pocket costs of birth registration. The analysis remains exploratory, but it provides hopefully useful insights about the likely benefits from various policies for increasing rates of birth registrations in countries such as Niger.

## Methods

### Study purpose

The objective of the paper is to analyze the correlates of birth registration in Niger using regression analysis, with a specific focus on the reasons declared by parents as to why they have not registered their children at birth. The availability of data on those reasons helps in assessing the role of various constraints faced by parents in registering their children, after controlling for a wide range of variables that may also affect birth registration. In turn, the results can help inform policy options to increase birth registrations. This is done through simulations of the potential effect on overall birth registration rates of solving the various obstacles leading to low birth registration rates.

### Data source

The data used are from the nationally representative ENISED survey (Etude Nationale d’Evaluation d’Indicateurs Socio-Economiques et Demographiques) implemented in Niger in 2015 by the National Statistical Office. The objectives of the survey were (a) to measure achievements towards achieving the Millennium Development Goals (MDG) and targets adopted in the 2012–2015 Economic and Social Development Plan (PDES in French), (b) to provide a baseline for the 2016–2020 PDES, and (c) to monitor progress towards the Sustainable Development Goals. The survey includes information among others on (i) the characteristics of dwellings; (ii) household members and population data; (iii) marriage and fertility; (iv) family planning; (v) maternal and child health; (vi) malaria; (vii) breastfeeding and nutritional status; (viii) under-5 mortality; (ix) maternal mortality; (x) birth registration; (xi) knowledge, attitudes, and practices of HIV/AIDS; (xii) justice, governance, and security; and (xiii) accessibility of selected services.

### Regression approach

Given that birth registration, the dependent variable, is a dichotomic variable, either a logit or probit regression could be used to assess the correlates of birth registration. This analysis relies on probit regressions, with the marginal effects at the mean of the sample reported together with their standard errors. These marginal effects represent the percentage point increase or decrease in the likelihood of birth registration associated with various variables.

As is the case for any cross-sectional analysis of this nature, the marginal effects represent associations as opposed to true causal effects that could be obtained through experimental or quasi-experimental approaches. Nevertheless, estimates show an order of magnitude for the potential effects of variables on the likelihood for children to be registered. The sample for analysis includes all children under age 5 in the survey for which information is available on their birth registration status. Sample weights are used when providing basic statistics.

The ENISED survey includes a range of variables that could affect the likelihood of birth registration. Apart from variables at the level of children and parents/households, the survey includes information on the reasons why parents have not registered their child. To capture local geographic effects affecting birth registration, a first approach consists of including the leave-out-mean (LOM) birth registration rate in the primary sampling unit in the independent variables. The LOM birth registration rate is computed for all other children in the primary sampling unit, but not the child being considered, to avoid issues of endogeneity. The LOM birth registration rate is thus a measure of the ease of birth registration at the local level based on the share of other children at the local level who have been registered. This variable is likely to capture mostly supply-side factors affecting birth registration rates, but it could also capture demand-side effects related, for example, at the local level to social norms or affordability issues.

This LOM birth registration rate variable essentially captures all local effects that matter for birth registration, whether this relates to the distance to registration offices or other factors. A second approach to look at the potential impact of local effects consists of relying on LOM estimates of the shares of parents mentioning various reasons for nonregistration. This approach provides more information since the magnitude of the impact of various reasons for nonregistration as declared by other households in the primary sampling unit can be used to assess the impact of various constraints at the local level on each household individually. The approach is not perfect since reasons mentioned by other households need not apply to any given individual household, but it provides an order of magnitude of the gains that could be achieved under various scenarios whereby obstacles to birth registration are being removed locally.

Note that birth registration rates tend to be higher among women who deliver their child in health facilities, while rates are lower for women delivering at home. This is also the case in Niger. Unfortunately, the data on the location of deliveries is available only for children born in the last 12 months, while the regression analysis is conducted for all children under 5 to have a larger sample size and give more time for the registration to be observed. Therefore, the variable where women deliver their children is not included in the regression analysis.

## Results

### Basic statistics

Table 1 provides estimates of birth registration rates, as well as the reasons why some children are not registered and other information about the birth registration process. Only slightly more than half (52.77%) of children under the age of 5 are registered. In urban areas, most children are registered, but in rural areas, less than half are registered.

**Table 1:** Birth registration rates, reasons for non-registrations, and registration process

	Rural	Urban	All
	Birth registration rate (%)		
Birth registration	47.7	83.7	52.8
Not registered	52.3	16.3	47.2
	Reasons for non-registration (%)		
Lack of availability	23.2	8.7	22.6
Remoteness	27.9	19.6	27.5
Lack of knowledge	29.9	30.3	29.9
No utility	14.3	29.5	15.0
Costs	4.8	11.9	5.0
All	100	100	100
	Time taken for birth registration (%)		
Less than 1 month	89.8	90.8	90.0
1 month	3.8	3.9	3.8
2 months	2.0	1.8	2.0
3 months	1.2	1.7	1.3
More than 3 months	3.2	1.8	2.9
Total	100	100	100
	Deadline for withdrawal of birth certificate (%)		
Less than 1 month	36.9	33.9	36.2
1 month	9.4	9.5	9.4
2 months	6.4	8.6	6.9
3 months	4.6	12.4	6.4
More than 3 months	5.4	9.9	6.4
Do not know	37.3	25.7	34.7
Total	100	100	100
	Costs of birth registration (% or FCFA)		
00 FCFA	58.8	75.9	62.6
[0–200]	17.7	3.3	14.5
[200–500]	12.0	5.0	10.5
[500–1000]	5.9	6.1	5.9
More than 1000	3.6	9.4	4.9
Do not know	2.0	0.4	1.6
Total	100	100	100
Mean	FCFA 234.9	FCFA 334.1	FCFA 257.6

Source: Authors’ estimation using ENISED data

The reasons invoked by parents for not registering their children include the lack of offices where birth registration can take place (22.57% nationally); the remoteness of registration offices—which means that the offices are far from where households live (27.50%); the fact that parents do not know that they should register their children (29.90%); the fact that parents think that birth registration is not useful (14.96%); and the cost of birth registration (5.07%). Note that the remoteness of offices is subjectively declared by households—it is not based on an actual measure of the distance or time it might take for households to reach those offices as such measures are unfortunately not available in the survey.

For those not registering their children, lack of availability and remoteness are cited more often in rural areas, while lack of utility and cost are cited more often in urban areas. As mentioned in the introduction, in recent years, major progress was made in reducing the distance to registration facilities. While acknowledging the benefits of this reform, estimates in Table 1 suggest that as of 2015, perceptions of a lack of centers or of the remoteness of existing centers (interpreted from the point of view of households as the centers being located far from where the households live) remained widespread.

Table 1 also suggests that when parents register their child, the process is fairly rapid, as prescribed by the law. The law stipulates that birth registration must take place within 30 days of birth (Law 2007–30, article 41), with this requirement being reduced to within 10 days of birth if the birth takes place in a health facility (Law 2007–30, article 42). In principle, a penalty is applied when a child is not registered, but this is not enforced. According to the survey, birth registration not being made for 1 month or more occurred with only 1 in 10 children registered. Once registered, parents typically get the birth registration certificate relatively quickly, but some parents do not know when they need to get it. One of the remaining issues is that parents need to be informed that they must pick up birth certificates at the primary or secondary registration centers because the certificates are not made available at auxiliary centers.

While cost is not the main obstacle to birth registration, according to Table 1, birth registration is not always free as it is supposed to be under the law. In rural areas, 41.24% of parents who registered their child said that they incurred a cost in doing so. In urban areas, the share of households paying for birth registration is smaller at just under one-fourth (24.1%). Among parents who incurred a cost when registering their child, the average payment was lower in rural areas at FCFA 234.94 versus FCFA 334.12 FCFA in urban areas. These costs may appear low, at less than US$1, but it may still be an issue for households, given that many live in poverty.

As background for the regression analysis, Table 2 provides information on the characteristics of the survey sample according to some of the main variables used in the regression analysis, as well as birth registration rates according to these characteristics.

**Table 2:** Sample characteristics and birth registration rates by selected characteristics

		Population shares (%)			Birth registration rates (%)		
		Rural	Urban	All	Rural	Urban	All
Sex of the child							
Male		49.6	52.2	50.0	48.6	85.9	54.1
Female		50.4	47.8	50.1	46.7	81.3	51.4
Location							
Urban		–	–	14.2	–	83.7	–
Rural		–	–	85.8	47.7	–	47.7
Region							
Agadez		1.5	8.4	2.5	29.7	87.9	58.0
Diffa		3.3	3.1	3.2	32.9	70.7	38.0
Dosso		11.8	6.4	11.0	65.1	81.6	66.5
Maradi		23.0	16.6	22.0	51.5	70.5	53.6
Tahoua		19.2	9.5	17.8	57.9	94.0	60.6
Tillaberi		16.5	6.6	15.1	47.4	76.8	49.2
Zinder		24.9	12.5	23.2	31.1	80.6	34.9
Niamey		0.0	37.0	5.3	-	89.7	89.7
Age of the child							
Less than 1 year old		19.2	20.4	19.3	51.2	84.7	56.2
1 year old		18.3	20.3	18.6	53.5	83.9	58.2
2 years old		19.5	18.3	19.3	45.7	84.5	50.9
3 years old		23.7	20.5	23.2	45.2	81.7	49.8
4 years old		16.4	17.6	16.5	42.0	83.7	48.3
Asset quintile							
Bottom		25.9	1.8	22.5	44.5	42.0	44.5
Second		23.5	3.1	20.6	41.2	56.4	41.5
Third		23.4	3.8	20.6	44.1	72.7	44.9
Fourth		21.6	25.5	22.1	54.8	74.2	58.0
Top		5.6	65.9	14.2	76.5	90.4	85.6
Education of the head							
No education		83.7	58.3	80.1	43.8	78.7	47.4
Primary (partial or completed)		10.8	18.9	12.0	65.9	90.7	71.5
Secondary (partial or completed)		5.1	16.6	6.7	69.0	88.6	75.8
Post-secondary (partial or completed)		0.3	6.2	1.2	94.7	95.8	95.6
Education of the spouse							
No education		87.4	60.5	83.6	43.9	77.8	47.4
Primary (partial or completed)		9.3	19.7	10.8	65.9	93.5	73.0
Secondary (partial or completed)		3.2	17.6	5.2	79.5	94.8	86.8
Post-secondary (partial or completed)		0.0	2.2	0.3	100.0	94.1	94.4
Spouses married as children							
None		10.1	36,00	13.8	54.7	86.2	66.4
One out of three		0.1	0.1	0.1	46.6	100.0	50.6
Half		4.6	5.7	4.7	51.8	88.3	58.1
Two out of three		0.6	0.0	0.6	52.0	100.0	52.1
All		84.5	58.2	80.8	46.1	82.8	49.9
Disability status of child							
No disability		98.4	98.6	98.4	47.8	83.9	52.9
One or more disabilities		1.6	1.4	1.6	42.4	65.3	45.2
Marital status of the head							
Never married		1.2	1.2	1.2	36.3	90.2	44.3
Married once		64.7	71.7	65.7	47.1	84.8	52.9
Polygamous		29.9	23.1	28.9	49.0	79.7	52.4
Separated, widow, or divorced		4.2	4.0	4.2	50.2	84.2	54.8
Occupation of the head							
Agriculture		77.4	15.5	68.6	46.4	69.3	47.2
Domestic work		4.4	4.3	4.4	39.7	50.3	41.2
Administration		1.3	19.0	3.8	89.9	94.4	93.1
Trade		7.3	30.4	10.5	59.9	84.1	69.8
Other occupation		8.2	27.7	11.0	43.5	88.7	59.7
No occupation		1.4	3.2	1.6	60.8	86.9	67.9

Source: Authors’ estimation using ENISED data

Consider first the characteristics of the population. Most of the population is rural (85.8%), and among urban households, more than a third live in the capital city of Niamey. The most populated region is Zinder, with 23.2% of the population, while the least populated region is Agadez, with 2.45%. Households are categorized according to five quintiles of wealth, with wealth measured through a factorial analysis of key assets owned by the households [19]. The first three quintiles (bottom, second, and third) essentially represent the population in poverty, given the high rates of poverty in the country, but many households in the top two quintiles (fourth and top) are not well-off either. Most households in the top quintile live in urban areas.

The vast majority of household heads and spouses have no education at all, especially in rural areas. While efforts related to Education for All and other international initiatives are paying off in terms of increasing school enrollment, most of the adult population did not benefit from such efforts at the time when they were of schooling age. For spouses, an additional variable of interest is whether they married as children, that is, before the age of 18. This is indeed the case for 84.54% of spouses (Niger is the country with the highest prevalence of child marriage in the world). Just under a third of rural heads of household are polygamous and the proportion is just under one-fourth in urban areas. Most household heads are engaged in agriculture, with trading being the second most important employment category.

Next, we consider birth registration rates. The rates are lower for girls than boys. They also vary by geographic location, with, for example, 9 in 10 children registered in Niamey and Tahoua versus only 1 in 3 registered in Zinder. Also, as expected, is the fact that birth registration rates are higher in the top quintile of wealth, especially in urban areas. In rural areas too, birth registration rates are much higher in the top two quintiles in comparison to the bottom three even if they do not reach the levels achieved in urban areas in those quintiles. There is also a clear difference in birth registration rates by the education level of the household head and the spouse, with higher educational attainment typically associated with higher birth registration rates, albeit with a few exceptions. Birth registration rates are also higher when the mother married at an adult age than when she did as a child. Finally, the occupation of the household head also shows a relationship with birth registration rates, for example with, as expected, much higher rates (93%) among household heads working in administration (mostly accounted for by the formal public sector). The question is whether all those associations remain when controlling for other variables affecting birth registration rates.

### Regression analysis

To measure the relationship between the various variables and the likelihood of birth registration while controlling for other factors affecting birth registration, probit regressions are estimated. Table 3 provides the results through marginal impacts at the mean of the sample. Two models are estimated: (1) a model with the LOM for birth registration to capture all local effects likely to be associated with supply-side issues but possibly also capturing some demand-side issues; and (2) a model with LOMs for each of the reasons mentioned by parents as to why they did not register their children, which helps in disaggregating local effects. In addition, a wide range of controls are included in the analysis, with the same controls used for both models. Both models are estimated separately for urban and rural areas, which is feasible thanks to the relatively large sample size (6106 children under the age of 5 in rural areas, and 1683 children in urban areas). Key findings are as follows.

**Table 3:** Probit regressions for the correlates of birth registration, marginal effects (dF/dX)

	Model 1		Model 2	
	Rural	Urban	Rural	Urban
Leave-out means				
Birth registration	0.9990***	0.3764***		
	(0.0300)	(0.0354)		
Lack of availability			− 0.9600***	− 0.2545**
			(0.0573)	(0.1110)
Remoteness			− 0.9740***	− 0.3512***
			(0.0437)	(0.0822)
Lack of knowledge			− 0.9144***	− 0.3126***
			(0.0494)	(0.0724)
No utility			− 0.8451***	− 0.5969***
			(0.0736)	(0.0808)
Costs			− 0.7562***	− 0.184
			(0.1082)	(0.1494)
Geographic location (reference is Niamey for urban areas)				
Agadez	Ref.	0.0516***	Ref.	0.0444**
		(0.0187)		(0.0191)
Diffa	0.0528	0.0033	0.0413	− 0.0132
	(0.0387)	(0.0251)	(0.0400)	(0.0288)
Dosso	0.1014***	− 0.0237	0.0508	− 0.0328
	(0.0373)	(0.0342)	(0.0382)	(0.0357)
Maradi	0.0712*	0.0141	0.0378	0.0017
	(0.0365)	(0.0249)	(0.0375)	(0.0260)
Tahoua	0.0871**	0.0335	0.0385	0.0347
	(0.0371)	(0.0383)	(0.0376)	(0.0378)
Tillaberi	0.0630*	− 0.0006	− 0.0066	− 0.0123
	(0.0360)	(0.0519)	(0.0373)	(0.0546)
Zinder	0.0234	− 0.0238	− 0.0204	− 0.0492
	(0.0367)	(0.0356)	(0.0374)	(0.0382)
Characteristics of the child				
Female	− 0.0374***	− 0.0268*	− 0.0320**	-0.0270*
	(0.0144)	(0.0144)	(0.0142)	(0.0143)
Age				
1 year old	0.0215	0.0012	0.0236	0.0021
	(0.0227)	(0.0211)	(0.0224)	(0.0209)
2 years old	− 0.0405*	− 0.0253	− 0.0342	− 0.0237
	(0.0220)	(0.0225)	(0.0217)	(0.0223)
3 years old	− 0.0922***	0.0206	− 0.0900***	0.0203
	(0.0213)	(0.0208)	(0.0211)	(0.0207)
4 years old	− 0.0853***	− 0.0058	− 0.0806***	− 0.0038
	(0.0232)	(0.0233)	(0.0229)	(0.0230)
Disability	− 0.0805	− 0.0077	− 0.0906	0.0129
	(0.0572)	(0.0720)	(0.0563)	(0.0678)
Characteristics of the household				
Number of children				
0–5 years old	− 0.0023	− 0.0031	− 0.0019	− 0.0046
	(0.0066)	(0.0076)	(0.0066)	(0.0075)
6–12 years old	− 0.0113**	0.0148**	− 0.0093*	0.0115*
	(0.0048)	(0.0061)	(0.0048)	(0.0061)
13–17 years old	0.0245***	− 0.0025	0.0204**	− 0.0008
	(0.0090)	(0.0090)	(0.0089)	(0.0090)
Spouse married as a child	0.0031	0.0082	0.0081	0.0069
	(0.0232)	(0.0169)	(0.0229)	(0.0170)
Education of the head				
Primary	0.0575**	0.0042	0.0660***	0.0005
	(0.0243)	(0.0199)	(0.0238)	(0.0199)
Secondary	0.0498	− 0.0282	0.0584*	− 0.0347
	(0.0355)	(0.0249)	(0.0352)	(0.0254)
Post-secondary	0.3242	0.0409	0.3829	0.0361
	(0.2545)	(0.0427)	(0.2398)	(0.0436)
Education of the spouse				
Primary	− 0.0014	0.0606***	0.0024	0.0613***
	(0.0259)	(0.0175)	(0.0255)	(0.0172)
Secondary	0.1627***	0.0735***	0.1599***	0.0738***
	(0.0501)	(0.0226)	(0.0490)	(0.0225)
Post-secondary		− 0.0258		− 0.0368
		(0.0662)		(0.0681)
Marital status of head				
Never married	− 0.0158	0.0114	− 0.0169	0.0042
	(0.0763)	(0.0739)	(0.0744)	(0.0759)
Polygamy	0.0053	0	0.0005	− 0.0013
	(0.0191)	(0.0204)	(0.0189)	(0.0204)
Wealth quintiles				
Second	− 0.0057	0.0261	− 0.0002	0.013
	(0.0207)	(0.0544)	(0.0205)	(0.0569)
Third	0.0074	0.007	0.0162	0.0003
	(0.0207)	(0.0512)	(0.0205)	(0.0534)
Fourth	0.0601***	0.0192	0.0604***	0.0218
	(0.0212)	(0.0450)	(0.0210)	(0.0459)
Top	0.2059***	0.1065*	0.1992***	0.1213**
	(0.0391)	(0.0560)	(0.0387)	(0.0590)
Occupation of head				
Domestic work	− 0.1695***	− 0.1479*	− 0.1612***	− 0.1042
	(0.0457)	(0.0816)	(0.0452)	(0.0780)
Administration	0.4017***	0.0088	0.3960***	0.0049
	(0.0857)	(0.0253)	(0.0854)	(0.0256)
Trade	0.0497*	− 0.0402**	0.0570**	− 0.0448**
	(0.0288)	(0.0181)	(0.0286)	(0.0182)
No occupation	0.0638	0.0212	0.0869	0.0239
	(0.0740)	(0.0399)	(0.0730)	(0.0387)
Occupation of spouse				
Domestic work	− 0.0015	0.0121	− 0.004	0.0164
	(0.0156)	(0.0182)	(0.0155)	(0.0181)
Administration	− 0.1127	0.0605	− 0.1296	0.0662*
	(0.1664)	(0.0398)	(0.1589)	(0.0380)
Trade	− 0.1005**	0.0396	− 0.0819*	0.0421
	(0.0467)	(0.0280)	(0.0459)	(0.0271)
No occupation	− 0.0794	− 0.0306	− 0.076	− 0.0323
	(0.0568)	(0.0332)	(0.0559)	(0.0332)
Number observations	6106	1683	6106	1683

Source: Authors’ estimation using ENISED data.

Note: *** denotes *p* value at or below 0.01, ** at or below 0.05, and * at or below 0.1.

Consider first the findings for child-level characteristics. There is evidence that girls are less likely to be registered at birth than boys, which may denote parental preferences for boys. Controlling for other variables, girls are less likely to be registered by about three percentage points in both models. There is a negative association in rural areas between the age of the child and the likelihood of birth registration. This may seem surprising but is likely related to the efforts undertaken by the government to increase birth registration rates in rural areas in the years preceding the survey. These efforts have led to higher birth registration rates among younger children. Whether a child has a disability or not does not seem to affect the birth registration rate.

Consider next the parental and household characteristics. A higher level of educational attainment for the spouse is associated in a statistically significant way with a higher likelihood of birth registration for a child at the primary and secondary level (the fact that the effect is not statistically significant at the post-secondary level may be due to small sample sizes at that level). Gains are also observed for the educational attainment of the head of household, but they tend to be smaller and less often statistically significant. A higher socioeconomic status, as measured by the quintiles of wealth, also is a positive factor, starting with the fourth quintile in rural areas and the fifth quintile in urban areas (recall that households in the bottom three quintiles tend to be poor).

Occupations matter as well, especially for the head of household, with domestic work associated with a lower likelihood of birth registration than the reference category of agricultural workers. In rural areas, being in administration is associated with a large increase in the likelihood of birth registration as was the case for basic statistics, but this is not the case in urban areas where birth registration rates are higher. Trading—which may denote a different status depending on whether the household is in rural or other areas, is associated with a gain in birth registration in rural areas and a loss in urban areas versus heads of the household involved in agriculture. Occupational effects for spouses tend not to be statistically significant with a few exceptions.

The marital status of the head of household, or the fact that the spouse married as a child, does not seem to have an effect. For the number of children in the household, effects in urban areas are negative for children aged 6–12 (possibly because of implications for standards of living or because the need to take care of those children reduces the time available to register children), while they are positive for older children (perhaps because some of the children are working and bringing in resources or because they can take care of younger siblings during parental absences). In urban areas, the effects are less likely to be statistically significant.

Consider finally findings for geographic effects at the level of regions as well as the effects of the LOM variables at the level of primary sampling units. First, there are differences in the likelihood of birth registration between regions after controlling for other variables that may affect birth registration, but these effects are observed mostly in rural areas, and many are not statistically significant. Second, as mentioned in the introduction, the LOM variables capture local effects affecting birth registration in different geographic areas. When the LOM for birth registration is used, it is likely to capture supply-side factors (when facilities for birth registration are too remote or not available at all), but it may also account for demand-side factors such as social norms or perceptions about the utility of birth registration. When the LOM variables for the obstacles to birth registration are used, the information provided is more granular.

In the first model, there are clear local effects at work, with the magnitude of the effects being larger in urban than in rural areas. In the second model, the role of different factors can be explored since effects are available by the type of factor that leads parents not to register their children. In rural areas, all LOMs have large effects, but the largest effects in terms of the size of the marginal effects are observed for lack of availability and remoteness, followed by lack of knowledge, lack of utility, and cost. In urban areas, the ranking is different, as expected. Perceptions of a lack of utility matter more when they are observed according to the magnitude of the marginal effects, followed by lack of knowledge, lack of availability, and remoteness, with the marginal impact for costs not being statistically significant.

Using the second model, predictions can be made as to the gains in birth registration rates that could be achieved if the obstacles mentioned by households were alleviated. Essentially, the simulations set at zero the proportion of households mentioning any specific constraint at the local level and rely on the estimates of marginal impacts to predict birth registration rates. The magnitudes of the gains thus depend on both the estimates of the marginal effects and the proportions of households citing each specific reason for not registering their children.

The results are provided in Table 4. The table first provides the observed and predicted birth registration rates under current conditions in urban and rural areas. The predicted rates are fairly close to the observed rates, which suggests that the model does a good job of predicting observed values. Next, Table 4 provides estimates of gains from solving the various issues mentioned by households as reasons for not registering their children. This is done separately for each reason, and then jointly for all reasons together. In rural areas, the largest gains would be observed from solving issues related to remoteness, lack of knowledge, and lack of availability of services for birth registration. The same holds for urban areas, but the gains in birth registration rates from solving those issues are much smaller, as expected. If all constraints mentioned by households were solved, the birth registration rate in rural areas could increase by 37.90 percentage points. For urban areas, the gain would be at 9.01 percentage points.

**Table 4:** Predicted birth registration rates under various assumptions for local effects (%)

	Rural		Urban	
	Birth registration rate	Gain versus base value	Birth registration rate	Gain versus base value
Observed value	47.7	–	83.7	–
Predicted value	47.1	–	83.8	–
No lack of availability	55.5	8.4	84.4	0.6
No remoteness	57.7	10.6	85.2	1.4
No lack of knowledge	57.6	10.5	86.4	2.6
No lack of utility	51.9	4.8	85.2	1.4
No issue of cost	48.6	1.4	84.0	0.2
All	85.0	37.9	92.8	9.0

Source: Authors’ estimation using ENISED data

## Conclusion

Despite major progress in recent years towards higher birth registration rates, almost one in two children may still not be registered at birth in Niger according to a recent nationally representative household survey. What can be done to improve birth registration rates? The factors leading to low birth registration are multiple, but the analysis of the ENISED survey provided in this paper suggests that some factors may play a larger role than others. Child-level factors, parental and household level factors, and local-level factors have been considered.

In terms of child-level characteristics, girls are less likely to be registered at birth than boys, even after controlling for a wide range of other factors that may affect birth registration. This may denote parental preferences for boys within the context of broader social norms that lead to multiple forms of disadvantage for girls and women, ranging from child marriage to lower levels of educational attainment, and lower earnings and labor force participation, than for men. Yet, while gender effects seem to be present, other factors have a larger effect on the likelihood of birth registration. Lack of educational attainment, especially for women, as well as lower levels of household wealth and less attractive employment for heads of household, are all associated with substantial reductions in the likelihood of birth registration for young children.

Still, the factors that tend to have the largest effect on birth registration are local effects related to whether services are available to register children in proximity to households, whether parents know that they should register their children, whether they are convinced that there are benefits of doing so, and whether they can afford the cost of birth registration. These effects were captured using LOM variables at the level of the primary sampling units where households live. Simulations based on the regression analysis suggest that in both rural and urban areas, the largest gains in birth registration rates would be observed from solving issues related to remoteness, lack of knowledge, and lack of availability of services for birth registration. However, as expected, gains would be much smaller in urban than in rural areas. Issues of cost, while important, play a smaller role in leading to nonregistration.

In a country as large as Niger, where population density is low, it should not come as a surprise that issues related to the lack of service availability or the remoteness of available services play an important role in the tendency for many children not to be registered at birth. But the approach used in this paper helps in quantifying those effects, while also pointing to other constraints, such as the fact that many parents do not know that they should register their children, or do not find it useful to do so. The policy responses to these various issues need to be varied and adapted to local realities. Different regions, or smaller geographic areas within broader regions, face different challenges. The good news is that information on the reasons leading parents to not register their children can be used to tailor appropriate interventions.

## Data Availability

Not applicable.

## Abbreviations

CRVS: Civil registration and vital statistics
ENISED: Etude Nationale d’Evaluation d’indicateurs Socio-Economiques et Demographiques
LOM: Leave-out-mean
MDG: Millennium Development Goals
SDG: Sustainable Development Goals
UN: United Nations
UNICEF: United Nations International Children’s Emergency Fund
WHO: World Health Organization
WBG: World Bank Group
